# Efficacy of the shoelace technique for extremity fasciotomy wounds due to compartment syndrome

**DOI:** 10.1186/s12891-023-06849-1

**Published:** 2023-09-04

**Authors:** Atsunori Onoe, Takashi Muroya, Yoshihiro Nakamura, Fumiko Nakamura, Takuma Yagura, Mari Nakajima, Masanobu Kishimoto, Kazuhito Sakuramoto, Kentaro Kajino, Hitoshi Ikegawa, Yasuyuki Kuwagata

**Affiliations:** 1 Department of Emergency and Critical Care Medicine, Kansai Medical University, Hirakata, Osaka Japan 573-1010; 2 Department of Orthopaedic Surgery, Kansai Medical University, Hirakata, Osaka Japan

**Keywords:** Compartment syndrome, Extremity fasciotomy wound, Shoelace technique, Wound management

## Abstract

**Background:**

The shoelace technique for compartment syndrome allows application of sustained tightening tension to an entire wound and intermittent tightening of the shoelace without requiring its replacement or anesthesia. We retrospectively evaluated the usefulness of the shoelace technique in the management of extremity fasciotomy wounds before and after its introduction in our institution.

**Methods:**

We targeted 25 patients who were diagnosed as having compartment syndrome and underwent extremity fasciotomy at our hospital from April 2012 to December 2021. The N group, comprising 12 patients treated without the shoelace technique, and the S group, comprising 13 patients treated with the shoelace technique, were compared retrospectively for each outcome.

**Results:**

There were no significant differences between the two groups in patient background. Compared with the N group patients, all of the S group patients avoided skin grafting (S group: *n* = 0, 0%; N group: *n* = 6, 50.0%; *p* < 0.01). However, there was no significant difference in the number of days to final wound closure (S group: 39.5 [IQR 24.3–58.0] days; N group: 24.0 [IQR 18.5–31.0] days, *p* = 0.06).

**Conclusions:**

We considered the shoelace technique to be a useful wound closure method for fasciotomy wounds caused by compartment syndrome because it can significantly reduce the need for skin grafting and tends to shorten the wound closure period.

## Introduction

Compartment syndrome results in increased intramuscular compartment pressure due to compression by the fascia and impairs microvascular blood flow. The main cause is ischemia–reperfusion injury, which occurs when an initial restriction of the blood supply to muscle during injury is followed by perfusion and concomitant reoxygenation. The muscles in the affected extremity may become edematous, resulting in the production of exudate and inflammatory responses [1]. Untreated, the syndrome can cause serious damage to the nervous and muscular tissue of the involved compartment, which might lead to permanent functional deficit of the involved limb. Therefore, it is important to perform extremity fasciotomy as soon as possible after the diagnosis of compartment syndrome to decrease the pressure in the intramuscular compartment [2–5].

For fasciotomy wounds, even after reduction of muscle swelling, simple closure is difficult due to skin retraction, and the classic management of fasciotomy wounds is skin grafting. However, this results in an unsightly appearance, hypesthesia of the skin graft area, and a long period of fasciotomy wound management [2–5]. In 1993, Harris first reported the shoelace technique for fasciotomy wounds caused by compartment syndrome and achieved delayed primary closure in all five treated cases [6]. The shoelace technique allows the application of sustained tightening tension to the entire wound and intermittent tightening without replacing the shoelace or requiring anesthesia [6]. In this study, we retrospectively evaluated the usefulness of the shoelace technique in the management of extremity fasciotomy wounds before and after its introduction in our institution.

## Methods

### Study design, setting, and population

We targeted 25 patients who were diagnosed as having compartment syndrome and underwent extremity fasciotomy at our hospital from April 2012 to December 2021. The N group, comprising 12 patients treated without the shoelace technique, and the S group, comprising 13 patients treated with the shoelace technique, were compared retrospectively for patient background and each study outcome. Cases of increased intramuscular compartment pressure due to hematoma formation were excluded. We introduced the management of fasciotomy wounds with the shoelace technique in November 2016. The study protocol was performed in accordance with the Declaration of Helsinki and was approved by the Ethics Committee of Kansai Medical University (approval no.: 2021355). The requirement for individual informed consent was waived in accordance with the Personal Information Protection Law and National Research Ethics Guideline in Japan.

### Wound management the shoelace technique in the S group

The shoelace technique was performed in the operating room about 48 h after the fasciotomy but was applied in some cases more than 48 h after the fasciotomy due to the patient’s general condition. Staples were placed about 1 cm from the fasciotomy wound edge to prevent the wound edge from curling inward and at 2–3 cm intervals along the edge of the fasciotomy wound. Double vessel loops were passed through these staples to cross the fasciotomy wound like a shoelace. The vessel loops were tightened to maintain uniform tension at each anchor point along the edge of the wound, and knots were placed at both ends of the vessel loops. This method allows for constant traction on the wound edge and permits constant tightening of the vessel loops until final wound closure is achieved. The use of vessel loops in the shoelace technique has the advantage of maintaining continuous gentle traction at the fasciotomy wound margin due to their elasticity [7]. Because reoperation would be needed if the vessel loop breaks during treatment, we usually use two vessel loops with this technique.

**Fig. 1:**
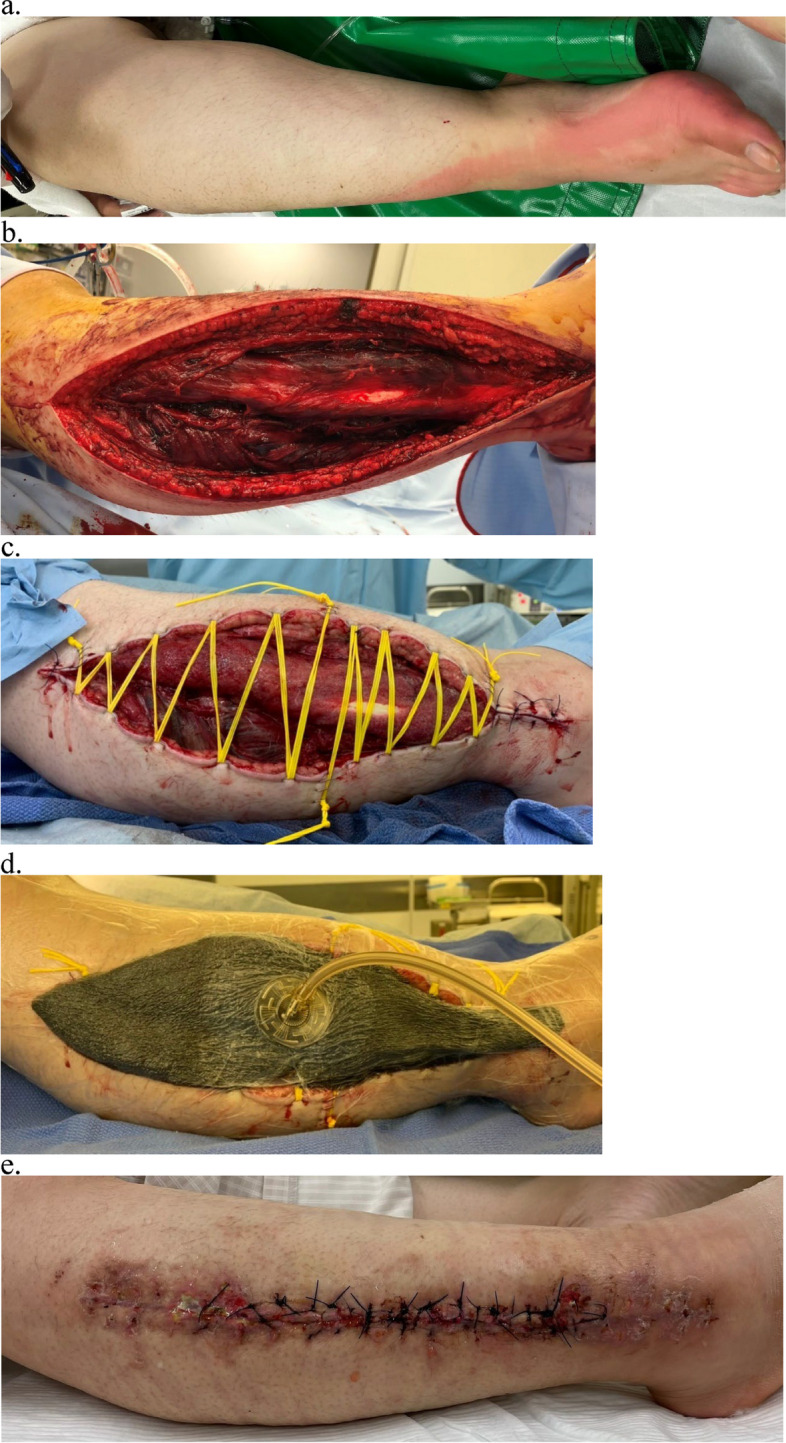
The patient, a 46-year-old female, had taken an overdose of sleeping pills and had lain in the same position for a long time, resulting in compression of the right lower leg (**a**). A right lower leg X-ray showed no fracture, but there was prominent swelling and pain in the right lower leg at the time of transport. The intramuscular compartment pressure in the right lower leg was elevated to more than 100 mmHg in all four compartments, which led to the diagnosis of compartment syndrome. We immediately performed fasciotomy for the four compartments via a single lateral lower leg incision (**b**). Forty-eight hours after the fasciotomy, we performed the shoelace technique (**c**) and placed the NPWT device (**d**). The shoelace was tightened twice a week, and the wound was completely closed on the 24th hospital day (**e**). *NPWT* negative pressure wound therapy

Negative pressure wound therapy (NPWT) was used after the shoelace technique was applied in cases of heavy effusion or severe muscle swelling. Thereafter, the shoelace was tightened at the bedside every 2–3 days. Wound care was performed by surgically draping the wound and disinfecting it. Finally, delayed primary closure was performed when the wound edges appeared sufficiently apposed to allow suturing without significant tension. We show a representative case of the shoelace technique in Fig. 1.

### Wound management in the N group

In patients in the N group, the fasciotomy wound was washed at the bedside with saline solution 2–3 times a week. Wound care was performed by surgically draping the wound and disinfecting it. If swelling was reduced and the edges of the fasciotomy wound could be sutured, several stitches were placed from the wound edge during wound care. The fasciotomy wound was then covered with gauze or the NPWT device. The wound was ultimately closed by delayed primary closure, skin grafting, and the creation of granulation and epidermis. If the fasciotomy wound was covered with something other than the NPWT device, wet gauze or gauze coated with povidone-iodine sugar ointment was selected according to the wound conditions. If the final skin defect was extensive, wound closure was performed with skin grafting. If the skin defect was small, the physician and patient conferred to determine whether to perform skin grafting or continue the wound closure procedure and wait for the creation of epidermis.

### Antibiotic administration and fracture treatment methods

After the extremity fasciotomy and surgical treatment of any fractures, cephazolin sodium was administered as an antibiotic for 48 h and antibiotic doses were adjusted according to renal function. However, if other infections, such as wound infection or pneumonia, complicated the treatment, appropriate antibiotics were administered according to the infection type.

When determining the time to surgically treat the fractures, the patient’s general condition and the condition of the soft tissue were taken into consideration. Depending on the fracture type, open-reduction and internal fixation were performed using plates, intramedullary nails, or other methods using Kirschner wires.

### Outcomes

The primary outcome was the avoidance of the skin grafting, and the secondary outcome was the time from fasciotomy to closure of the fasciotomy wound.

### Statistical analysis

Categorical data are presented as number (%) and were compared by the χ^2^ test or Fisher’s exact test as appropriate. The normality of items under consideration was tested with the Shapiro–Wilk test. Comparison of results between the two groups was done with the Student *t*-test for normally distributed items and the Mann–Whitney test for non-normally distributed items. Normally distributed data are reported as the mean (SD), whereas non-normally distributed data are reported as the median (interquartile range [IQR]). All tests were 2-tailed, and P values of < 0.05 were considered statistically significant. All statistical analyses were performed with use of the SPSS statistical package ver. 23.0 J (SPSS, Inc., Chicago, IL).

## Results

Twenty-five patients were enrolled in the study, and their characteristics are shown in Table 1. Their mean age was 43.0 ± 20.6 years in the N group and 50.7 ± 16.0 years in the S group. Eight patients (66.7%) in the N group were male, as were 11 (84.6%) patients in the S group. There were no significant differences between the two groups in any of the characteristics, including past medical history, complications of infection, and maximum muscle compartment pressure that might affect the fasciotomy wound. In the S group, the shoelace technique was performed on the 2nd day after fasciotomy in 2 cases (15.4%), 3–5 days after in 6 cases (46.2%), and on the 6th day or later in 5 cases (38.5%) (Table 1).

**Table 1:** Patient background

Background data	N group (*n* = 12)	S group (*n* = 13)	*P* value
Age (years), mean (S.D.)	43.0 ± 20.6	50.7 ± 16.0	0.31
Male, n (%)	8 (66.7)	11 (84.6)	0.28
ISS (points), median (IQR)	4.0 (4.0–7.8)	4 (4.0–4.0)	0.73
Diabetes, n (%)	0 (0)	2 (15.4)	0.17
Immunodeficiency, n (%)	0 (0)	0 (0)	―
Steroid user, n (%)	0 (0)	0 (0)	―
Total protein (g/dL), mean ± S.D	7.1 ± 0.7	7.0 ± 0.4	0.61
Albumin (g/dL), mean ± S.D	4.3 ± 0.6	4.4 ± 0.4	0.58
Body mass index (kg/m^2^), mean ± S.D	22.5 ± 5.0	23.3 ± 2.8	0.63
Injury location (forearm/lower leg), n (%)	5 (41.7)/7 (58.3)	6 (46.2)/7 (53.8)	0.57
Injury mechanism (trauma/crush), n (%)	6 (50.0)/6 (50.0)	7 (53.8)/6 (46.2)	0.85
Complications of fracture, n (%)	5 (41.7)	3 (23.1)	0.29
Maximum muscle compartment pressure (mmHg), median (IQR)	74.0 (51.0–77.0)	63 (53.0–93.5)	0.82
Days from transport to osteosynthesis, median (IQR)	1.5 (0.8–1.5)	9.0 (2.0–15.0)	0.08
Use of NPWT, n (%)	9 (75.0)	10 (76.9)	0.64
Days from fasciotomy to performing			
shoelace technique			
Day 2, n (%)		2 (15.4)	
Days 3–5, n (%)		6 (46.2)	
Day ≥ 6, n (%)		5 (38.5)	
Time from onset to incision (min), mean ± S.D	887.5 ± 692	682.3 ± 480	0.40
Neurological disorder, n (%)	7 (58.3)	7 (53.8)	0.82
Complications of infection, n (%)	4 (33.3)	1 (7.7)	0.14

*S.D* Standard deviation, *ISS* Injury Severity Score, *IQR* interquartile range, *NPWT* negative pressure wound therapy, *N group* patients treated without the shoelace technique, *S group* patients treated with the shoelace technique

All patients in the S group avoided skin grafting, resulting in a significant difference between the number of patients requiring a skin graft in the N group (S group: *n* = 0, 0%; N group: *n* = 6, 50.0%; *p* < 0.01). However, there was no significant difference in the number of days to final closure (S group: 39.5 (IQR 24.3–58.0) days; N group: 24.0 (IQR 18.5–31.0) days, *p* = 0.06) (Table 2).

**Table 2:** Outcomes

Outcome	N grou (*n* = 12)	S group (*n* = 13)	*P* value
Skin grafting, n (%)	6 (50.0)	0 (0)	< 0.01
Days from incision to wound closure, median (interquartile range)	39.5 (24.3–58.0)	24.0 (18.5–31.0)	0.06

*N group* patients treated without the shoelace technique, *S group* patients treated with the shoelace technique

## Discussion

We have shown in this study that the use of the shoelace technique for fasciotomy wounds caused by compartment syndrome can avoid the need for skin grafting and shorten the time required for closure of fasciotomy wounds. As there has been no consensus on an optimal technique for fasciotomy wound closure [8], our results may help surgeons realize the efficacy of the shoelace technique for the management of fasciotomy wounds.

There are various methods of fasciotomy wound management, and the recent introduction of NPWT has facilitated wound management. However, about 20% of NPWT-only cases required skin grafting. Pain and other sequelae occurred in 95% of cases in which skin grafting was performed, and in some cases, additional cosmetic surgery was performed [9, 10]. In our institution, we were able to close the wound in all cases, without any skin grafting, using both the shoelace technique and NPWT and the shoelace technique only within about 26 days after the fasciotomy. Johnson et al. report that NPWT alone is more likely to require skin grafting and that skin grafting increases the length of hospitalization and medical costs [11]. The material used in the shoelace technique is readily available in any standard operating theater, making this procedure also useful in countries with limited resources [12]. The mean daily cost of treatment for each patient managed by the shoelace technique was reported to be as low as about 14 Euros [9]. The use of the shoelace technique is expected to contribute to shorter hospital stays and lower medical costs by avoiding the need for skin grafting and shortening the closure period of fasciotomy wounds.

Eid and Elsoufy performed the shoelace technique in 17 patients with fractures and reported no major complications [12]. In the present study, 3 patients with fracture complications were included in the S group. The shoelace technique was performed on the fasciotomy wounds in all 3 of these patients, and they all progressed without problems.

The median days from incision to wound closure in the S group was 24.0 (IQR 18.5–31.0) days, whereas that in the shoelace groups in previous studies ranged from 7.6 to 15.1 days, shorter than that in the present study [9, 11]. However, these previous studies did not describe elevated intramuscular compartment pressures. Therefore, it is not possible to simply compare the days from incision to wound closure in this study with these previous studies. The median maximum muscle compartment pressure in the S group was 63 (IQR 53.0–93.5) mmHg, which may have delayed the days from incision to wound closure due to highly elevated intramuscular compartment pressure. Dodenhoff and Howell used the shoelace technique at fasciotomy and reported that the fasciotomy wound could be gradually closed by tightening the vessel loops from 48 h after the fasciotomy with attention to any increase in intramuscular compartment pressure [11, 13]. In our institution, fasciotomy only was performed on the day of diagnosis of compartment syndrome. Because of the severe muscle swelling after fasciotomy, we attempted to perform the shoelace technique at about 48 h after the fasciotomy in the operating room. However, Dodenhoff and Howell did not recommend the use of the shoelace technique if the intramuscular compartment pressure continues to increase at 48 h after fasciotomy [13]. In the S group, the shoelace technique was performed at 48 h after fasciotomy in only 2 patients (15.4%) according to the patient’s general condition and wound swelling at 48 h after fasciotomy. When compared to the other studies, these factors may have affected the delay in days from incision to wound closure.

This study confirmed the usefulness of the shoelace technique as a management method for fasciotomy wounds.

### Limitations

The limitations of this study are first, its small sample size and that the volume of intravenous infusion during treatment, which may affect the degree of swelling at the fasciotomy site, is unknown. Second, this was a retrospective study. Third, in the N group, if the skin defect was small, the physician and patient conferred to determine whether to perform skin grafting or continue the wound procedure and wait for the creation of epidermis. This could have affected the number of days from incision to wound closure.

## Conclusions

We investigated the usefulness of the shoelace technique for fasciotomy wounds caused by compartment syndrome. We consider the shoelace technique to be a useful wound closure method for fasciotomy because it can significantly reduce the use of skin grafting and tends to shorten the wound closure period.

## Data Availability

The datasets used and/or analyzed during the current study are available from the corresponding author on reasonable request.

## Abbreviation

NPWT: Negative pressure wound therapy
